# Application of Excimer Lamp in Quantitative Detection of SF_6_ Decomposition Component SO_2_

**DOI:** 10.3390/s21248165

**Published:** 2021-12-07

**Authors:** Tunan Chen, Kang Li, Fengxiang Ma, Xinjie Qiu, Zongjia Qiu, Zhenghai Liao, Guoqiang Zhang

**Affiliations:** 1 Institute of Electrical Engineering, Chinese Academy of Sciences, Beijing 100190, China; tnchen@mail.iee.ac.cn (T.C.); likang07@mail.iee.ac.cn (K.L.); qiuzongjia@mail.iee.ac.cn (Z.Q.); liaozhenghai@hotmail.com (Z.L.); 2 University of Chinese Academy of Sciences, Beijing 100049, China; 3 Electrical Power Research Institute, Anhui Electrical Power Co., Ltd., State Grid, Hefei 230601, China; njumfx@hotmail.com (F.M.); sgccsf6@hotmail.com (X.Q.)

**Keywords:** SF_6_-insulated equipment, excimer lamp, absorption spectroscopy, online monitoring

## Abstract

Accurate quantitative detection for trace gas has long been the center of failure diagnosis for gas-insulated equipment. An absorption spectroscopy-based detection system was developed for trace SF_6_ decomposition SO_2_ detection in this paper. In order to reduce interference from other decomposition, ultraviolet spectrum of SO_2_ was selected for detection. Firstly, an excimer lamp was developed in this paper as the excitation of the absorption spectroscopy compared with regular light sources with electrodes, such as electrodeless lamps that are more suitable for long-term monitoring. Then, based on the developed excimer lamp, a detection system for trace SO_2_ was established. Next, a proper absorption peak was selected by calculating spectral derivative for further analysis. Experimental results indicated that good linearity existed between the absorbance and concentration of SO_2_ at the chosen absorption peak. Moreover, the detection limit of the proposed detection system could reach the level of 10^−7^. The results of this paper could serve as a guide for the application of excimer lamp in online monitoring for SF_6_-insulated equipment.

## 1. Introduction

Sulfur hexafluoride (SF_6_) possesses great insulation capabilities and arc extinction ability. Since 1960s, SF_6_ has been applied in many gas-insulated equipment such as transformer, inductor, GIS (gas-insulated switchgear), GIL (gas-insulated transmission line) and so on [1]. With the development and popularization of SF_6_-insulated equipment, the insulation state detection for it has long been the center of research field. Based on current studies, various detection techniques have been presented. For instance, ultra-high frequency (UHF) [2], frequency-domain dielectric spectroscopy (FDS) [3], ultrasonic method [4] and so on were proposed to detect relevant electrical parameters. However, such detection techniques were vulnerable to electromagnetic interference or vibration noise. Moreover, such invasive detections could impair the intact structure of SF_6_-insulated equipment and result in leakage. Therefore, it is necessary to find a non-electrical method to properly evaluate the insulation state of SF_6_-insulated equipment.

Decomposition gas analysis is one of the prevailing non-electrical methods for estimating the insulation state of electrical equipment. Even though SF_6_ itself is a colorless, odorless and innocuous inert gas, its decompositions under discharge are poisonous and corrosive. Specifically speaking, under the condition of discharge, SF_6_ will decompose and generate a series of sulfide. Furthermore, some of the sulfide will react with micro water and oxygen in the equipment to generate H_2_S, SO_2_, SOF_2_, SO_2_F_2_ and so on [5]. To this day, plenty of techniques have been developed to detect trace SF_6_ and its decompositions [6,7]. Moreover, some forms of commercial products are also available [8,9].

Meanwhile, plenty of research studies have verified that among all the decomposition of SF_6_, the concentration of SO_2_ strongly correlates with the discharge level inside the equipment [10]. The larger the concentration, the higher the discharge level. In that case, detecting the concentration of the decomposition of SF_6_, especially SO_2_, has become a widely acceptable method for monitoring the insulation state of SF_6_-insulted equipment. Among all the detection method for SO_2_, optical techniques have become more and more prevalent due to its short response time and high accuracy.

Nowadays, common optical techniques for trace SO_2_ detection include differential optical absorption spectroscopy (DOAS) [11], photoacoustic spectroscopy (PAS) [12], fluorescence spectroscopy [13], Fourier transform infrared spectroscopy (FTIR) [14], et cetera. Among those methods, absorption spectroscopy possesses several merits such as short response time, simple structure and high accuracy. In addition, compared with other optical detection techniques, the detection system based on absorption spectroscopy is more suitable for modularized and portable design. Therefore, absorption spectroscopy is extensively applied in the electrical industry for insulation state detection.

Usually, the detection system for SO_2_ based on absorption spectroscopy employs infrared excitations. However, SO_2_ possesses two strong absorption bands in ultraviolet (UV) range. According to Beer–Lamber’s law, the larger absorption cross-section band of SO_2_ contributes to better detection performance. Moreover, the majority decomposition of SF_6_ except for SO_2_ has no evident absorption band in the UV range. Hence, the employment of UV excitation is able to avert crossover interference to a great extent, which results in more accurate spectral information.

Usual UV excitation includes deuterium lamp [15], xenon lamp [16] and neon lamp [17]. However, such excitation depends on an electrode to launch electron and excite gas discharge. Exposed to long-term UV illumination, the ageing process of an electrode is accelerated. On the other hand, the excimer lamp, which does not have an electrode, excites gas to discharge using microwave. Due to this characteristic, the lifetime of the excitation drastically improved. Accordingly, the frequency of systematic maintenance is reduced. As a consequence, the detection system based on excimer lamp is feasible for long-term monitoring scenarios.

Aiming at the discussion above, a trace SO_2_ quantitative detection system based on excimer lamp is presented in this paper. The major contributions of this paper are as follows:

(1) Study of the characteristic of UV excimer lamp and the feasibility of its application in UV absorption spectroscopy;
(2) Establishment of trace SO_2_ quantitative detection system based on excimer lamp and evaluation of the performance of the presented detection system;
(3) Selection of the most prominent absorption peak among the absorption spectra by calculating spectral derivative for quantitative analysis.

The result of this paper could serve as a reference for the application of excimer lamp in the field of gas insulated equipment fault diagnosis.

## 2. Theoretically Fundamental

### 2.1. Basic Principle of Excimer Lamp

Generally, excimer includes noble gas, halogen, noble gas-halogen and mercury-halogen dimer [18]. Noble gas–halogen is the most common working substance of an excimer lamp. The working principle of such an excimer lamp could be demonstrated as follows.

Excited by energetic electron, noble gas and halogen are ionized, and their processes are described as follows [19]:

(1) $$e^{*}+Rg\to Rg^{*}+e$$

(2) $$e^{*}+Rg\to Rg^{+}+2e$$

(3) $$e^{*}+X_{2}\to X+X^{-}$$

where Rg represents noble gas particle, and X represents halogen particle. Then, the excimer is generated through a Harpooning reaction:

(4) $$Rg^{+}+X^{-}+M\to RgX^{*}+M$$

(5) $$Rg^{*}+X_{2}\to RgX^{*}+X$$

where M represents three kinds of particle which include the atom, molecule and buffer gas. Such an excimer is not stable; it will decompose and release excitation energies through photons:

(6) $$RgX^{*}\to Rg+X+h\nu$$

where h represents the Planck constant, and ν represents the wavelength of the photon.

In this paper, a microwave was employed to bring electron kinetic energy and excite the working substance in the excimer lamp. Then, photons with certain wavelength could be obtained.

### 2.2. Basic Principle of Absorption Spectroscopy

According to Beer–Lambert’s Law, the absorption spectrum could be expressed as follows [20].

(7) $$I\left(\lambda \right)=I_{0}\left(\lambda \right)\exp\left[-cL\sigma \left(\lambda \right)\right]$$

Equation (7) can be rewritten as follows:

(8) $$c=\frac{\ln\left[\frac{I_{0}\left(\lambda \right)}{I\left(\lambda \right)}\right]}{L\sigma \left(\lambda \right)}=\frac{A\left(\lambda \right)}{L\sigma \left(\lambda \right)}$$

where $I_{0}\left(\lambda \right)$ represents the initial intensity of UV light, I(λ) represents the transmission intensity of UV light, L represents the optical path length, σ(λ) represents the absorption cross-section of the investigated gas, c represents the concentration of investigated gas and λ represents the wavelength of incident light. In addition, absorbance is denoted as $A\left(\lambda \right)=\ln\left[I_{0}\left(\lambda \right)/I\left(\lambda \right)\right]$.

## 3. Experimental Setups

To quantitatively detect trace SO_2_, a detection system based on absorption spectroscopy was established in this paper. Crucial components of the detection system were introduced as follows.

### 3.1. The Selection of the Formula of Excimer Lamp

In order to select a proper excitation for the detection system, the absorption characteristic of SO_2_ was studied first in this paper. Based on current studies, it was recognized that two major absorption bands of SO_2_ exist in UV band. According to the principle of absorption spectroscopy, the nominal wavelength of the excitation falling into the absorption band of investigated gas contributes to better performance of the detection system. Given the data provided by Gordon [21] and Blackie [22], the absorption cross section of SO_2_ in the UV band can be depicted as follows.

**Figure 1:**
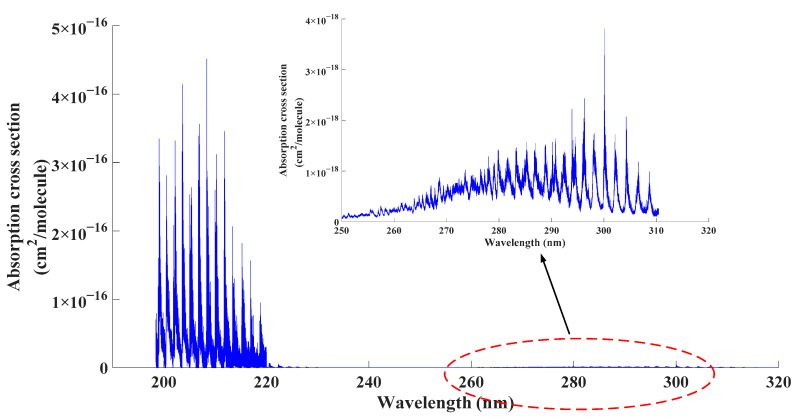
Absorption cross section of SO_2_ in UV range.

From Figure 1, according to current research studies [23,24], in the wavelength range between 198 nm and 310 nm, SO_2_ exhibited two strong absorption band, which were at 190 nm to 220 nm (^1^*B*_2_←^1^*A*_1_) and 250 nm to 310 nm (^1^*A*_2_, ^1^*B*_1_←^1^*A*_1_). Compared with the absorption band at 250 nm to 310 nm, the absorption band at 190 nm to 220 nm was one order of magnitude larger. In that case, it would be better if the nominal wavelength of employed excimer lamp was in the range of 190 nm to 220 nm.

Former scholars have performed abundant research on the characteristics of various excimer lamps. According to reference [25], regular formulas of noble gas–halogen excimer lamp and corresponding nominal wavelength are described as follows.

From Table 1, combined with the absorption band of SO_2_, a I-Kr lamp was selected as the excitation in this paper.

After the working substance has been selected, the corresponding contents were determined then. The luminance of excimer lamp relies on the gas discharge inside the lamp. On the one hand, light intensity was determined by the number of excited gas molecules. On the other hand, based on the theory of gas discharge, the mean free path of an electron was described as follows:

(9) $$\bar{\lambda }=\frac{1}{\pi N{\left(r_{1}+r_{2}\right)}^{2}}$$

where $\bar{\lambda }$ represents the mean free path of electron, N represents the density of gas molecule $r_{1}$ and $r_{2}$ represent the radius of electron and gas molecule, respectively. From (9), higher pressure results in higher densities and smaller mean free path. Moreover, a smaller mean free path indicates less time to gain energy for an electron. Hence, the probability of gas molecules being ionized decreases accordingly. From a macro point of view, the emission intensity of an excimer lamp decreases. To conclude, the light intensity might increase first and decrease later when the content of I_2_ increases.

**Figure 2:**
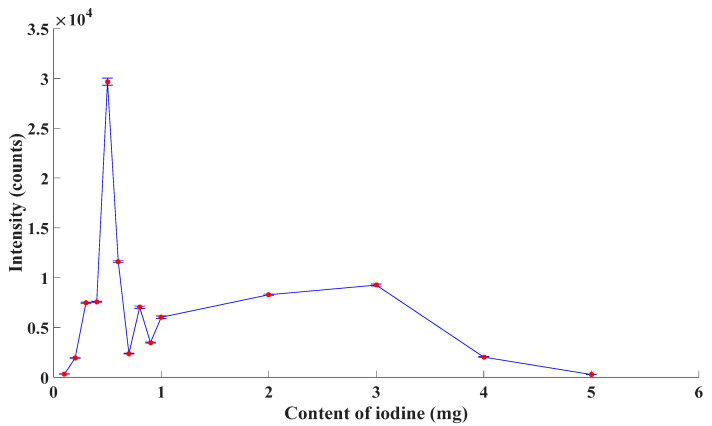
Emission intensity of excimer lamps.

In order to verify the theory aforementioned and to select the proper formula of the excitation, in this paper, the emission spectroscopy of excimer lamps with different content of I_2_ was detected. In those excimer lamps, the content of I_2_ was 0.1 mg, 0.2 mg, 0.3 mg, 0.4 mg, 0.5 mg, 0.6 mg, 0.7 mg, 0.8 mg, 0.9 mg, 1 mg, 2 mg, 3 mg, 4 mg and 5 mg. As the ambient gas, the pressure of Kr was 2 Torr. The experimental results are described as Figure 2.

From Figure 2, the emission intensity of excimer lamps increased first and decreased later with an increase in the content of I_2_ roughly, which agreed with the statement above. The emission intensity reached a peak when the content of I_2_ was 0.5 mg. Thus, combined with both theoretical analysis and experimental outcome, the content of I_2_ was chosen as 0.5 mg in this paper.

### 3.2. Basic Structure of Detection System

According to the basic principle of absorption spectroscopy, a trace SO_2_ detection system was built in this paper. The schematic diagram of the detection system is described as follows.

**Figure 3:**
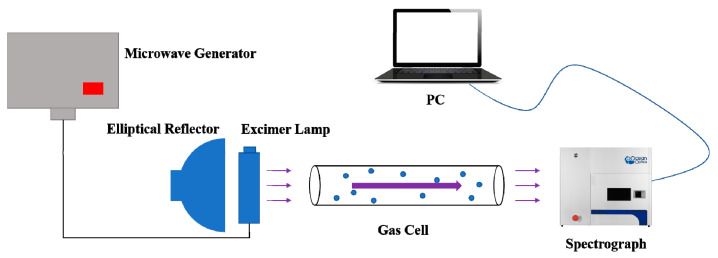
Schematic diagram of the detection system.

From Figure 3, the optical path length of the gas cell was 0.8 m, and the model of the spectrometer was OceanOptics MX2500+. A microwave generator comprised a transformer and a magnetron. A generated microwave was transmitted to an excimer lamp through a cable. The gas molecules were excited by the microwave and generated UV light, which entered the gas cell and was absorbed by the investigated gas. The elliptical reflector behind the excimer lamp was employed to enhance light intensity of the investigated gas. Then, the transmission light arrived at the probe of the spectrometer for detection. Finally, data obtained from the spectrometer were transmitted to a PC for further processing.

## 4. Experimental Results and Analysis

### 4.1. The Influence of Dark Current

Considering the interference from surrounding and background noise, the spectrometer would capture signals without the excitation being started. It is necessary to eliminate such influence before spectral data are further processed. Denote such influence as dark current, the modified absorbance is then calculated as follows:

(10) $$A\left(\lambda \right)=\ln\frac{I_{0}\left(\lambda \right)-I_{N}\left(\lambda \right)}{I\left(\lambda \right)-I_{N}\left(\lambda \right)}$$

where A(λ) represents the absorbance of investigated gas at wavelength λ, $I_{0}\left(\lambda \right)$ represents the initial intensity of UV light, I(λ) represents the transmission intensity of UV light and $I_{N}\left(\lambda \right)$ represents the dark current.

### 4.2. Quantitative Analysis of Trace SO_2_ Detection

**Figure 4:**
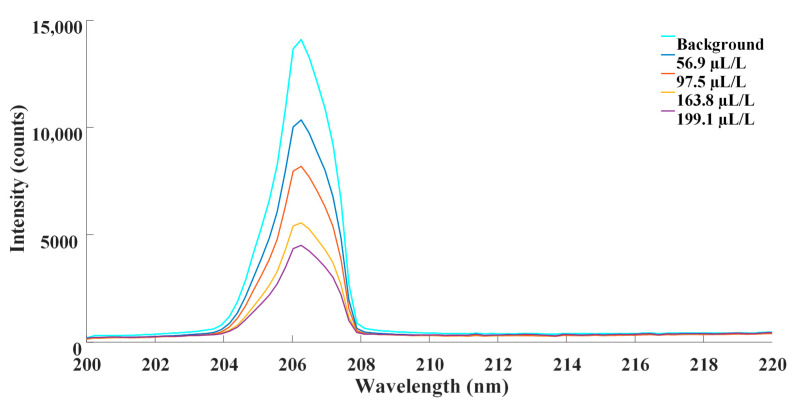
Absorption spectra of transmission at different concentration of SO_2_.

Standard SF_6_ gas of 99.999% purity was employed as the background gas. SO_2_ gas samples with the concentration of 0 μL/L, 56.9 μL/L, 97.5 μL/L, 163.8 μL/L and 199.1 μL/L were taken for experiment. SO_2_ with the concentration of 0 μL/L, which was pure SF_6_, was considered as the background. Gas samples were prepared by mixing certain volumes of standard SO_2_ gas with known concentrations and pure SF_6_. A spectrometer was used to detect the absorption spectra. Then, the dark current was deducted from detected data. With background subtraction, the modified absorption spectra of the aforementioned concentrations are depicted as Figure 4.

In order to determine the most prominent absorption peak among all wavelengths, spectral derivatives were obtained. By calculating the derivatives of the absorbance spectra, matching between the absorption cross section of the investigated gas and the emission spectrum of the excitation could be quantitatively determined. Therefore, the most prominent absorption peak could be located. Such processing had potential for various objects and scenarios instead of certain kinds of investigated gas.

**Figure 5:**
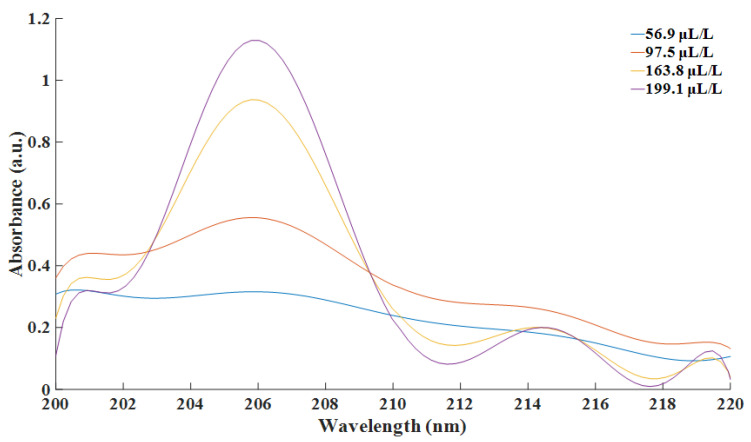
Smoothed spectra at different concentration of SO_2_.

In this paper, Savitzky–Golay convolution filtering was employed to smooth the original spectral data and to eliminate irrelevant noise [26]. The order of the Savitzky–Golay filter was set at nine. The filtered spectra are depicted as Figure 5.

Then, one-order derivative spectra of the absorbance at each concentration of SO_2_ were calculated and depicted as follows.

**Figure 6:**
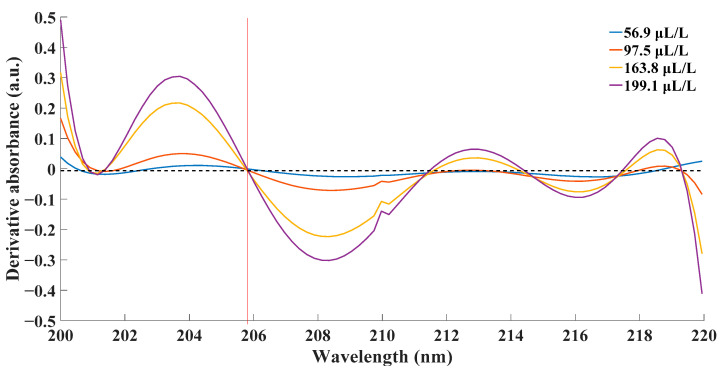
Derivative spectra of absorbance at different concentration of SO_2_.

**Figure 7:**
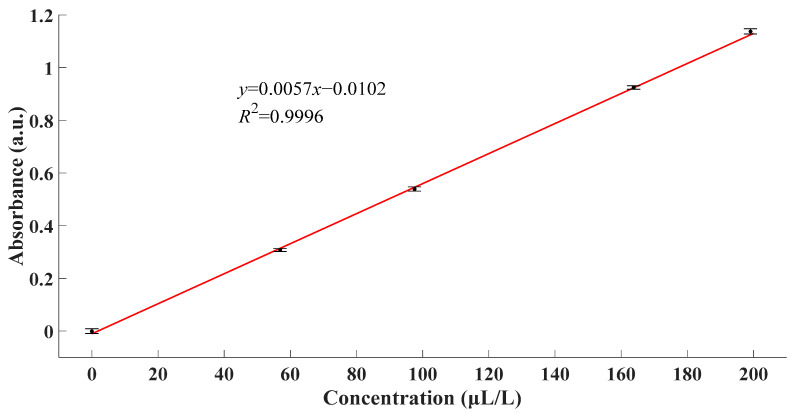
Linear fitting between absorbance and concentration.

From Figure 6, it could be observed that the zeros of four derivative absorbance spectra met at the point of 205.79 nm. The fact indicated that all four spectra reached their extremums at this very point. Combined with original spectra, the absorbance at this point was the maximum. According to the theory of absorption spectroscopy, a more prominent phenomenon contributed to better performance of the detection system. Ergo, the experimental data at the wavelength of 205.79 nm were used for further calculation and analysis. Linear fitting between absorbance and concentration at this wavelength was as Figure 7.

From Figure 7, linear fitting *R*^2^ > 0.9996 confirmed the linearity of the absorbance response to the concentration of SO_2_. Moreover, the linear fitting function could be expressed as follows.

(11) A=0.0057c−0.0102

From what has been discussed above, the fitting result proved the rationality of using a linear function to characterize the relationship between the absorbance and the concentration of SO_2_. As a result, SO_2_ gas with unknown concentrations could be calculated by (11) once the corresponding absorbance was measured.

### 4.3. Estimation of the Detection Performance

In this paper, the performance of the proposed detection system was evaluated from three aspects, accuracy, DL and drift over time, which are demonstrate successively.

Firstly, the accuracy of the proposed detection system was quantitatively estimated. In detail, in order to evaluate the accuracy of the proposed detection system, gas samples with different concentrations of SO_2_ were employed as the benchmark for comparison. Gas samples were prepared by mixing certain volumes of standard SO_2_ gas with known concentrations and pure SF_6_. Standard gas was purchased from Beijing Haipubeifen gas product company. Based on the error of standard gas and barometer employed in this work, the error value of the concentrations of gas samples was ±2.255%. The main process of obtaining detection results was as follows:

(1) Introduce one gas sample into the gas cell for detection;
(2) Detect five successive points of transmission light intensity and calculate their average as the detection transmission light intensity;
(3) Take the detection transmission light intensity to calculate the corresponding concentration. The calculated concentration is then taken as the detection result;
(4) Clean the detection system and repeat above procedure in order to obtain the detection result of each gas sample.

By comparing the detection result with the corresponding gas sample, the relative error could be obtained. Hence, the accuracy of proposed detection system could be evaluated. Furthermore, in order to fully evaluate the performance of the proposed detection system, the error budget was presented. In this study, the error mainly came from the error of standard gas, barometer and detection process. In detail, the error of purchased standard gas was 2%, and the error level of the barometer was 0.25. Therefore, the gas samples prepared in this study had an error of ±2.255%. Moreover, the errors of experimental data obtained from spectrometer were evaluated by calculating the standard deviation of raw data, which are given next. Finally, the comparison results are listed as follows.

From Table 2, it could be concluded that the proposed detection system could measure trace SO_2_ with high accuracy at both low and high concentrations. Such capability brought the applicative potential to the proposed detection system.

Secondly, the detection limit (DL) is a common criterion employed to gauge the capability of a detection system. The DL (1σ) of the presented detection system was calculated as follows [27]:

(12) $$DL=\frac{c}{SNR}$$

where c represents the concentration of SO_2_, and SNR represents the signal to noise ratio.

In this paper, the main process of obtaining DL was as follows:

(1) Introduce pure SF_6_ into the gas cell for detection;
(2) Detect five successive points and calculate their average as the background signal and the standard deviation of their absorbance as the systematic noise;
(3) Clean the detection system and introduce gas samples into the gas cell for detection;
(4) Detect five successive points of transmission light intensity and calculate the average of their absorbance;
(5) Calculate DL.

Based on the aforementioned procedure, the performance of the proposed detection system could be evaluated. Particularly speaking, the systematic noise represented the fluctuation degree of obtained data from the spectrometer and reflected the uncertainty of calculation results. A gas sample with the concentration of 56.9 μL/L was taken to calculate SNR and DL. The calculation results are listed as follows.

From Table 3, low systematic noise indicated that the emission of the excitation was stable, which was beneficial to high SNR. Accordingly, low DL could be obtained. It could be observed that the DL (1σ) of the detection system was 0.632 μL/L. Currently, the SO_2_ diagnostic threshold level is about 1 μL/L. Therefore, the results proved the feasibility of the application of proposed detection system in failure diagnosis in SF_6_-insulated equipment.

**Figure 8:**
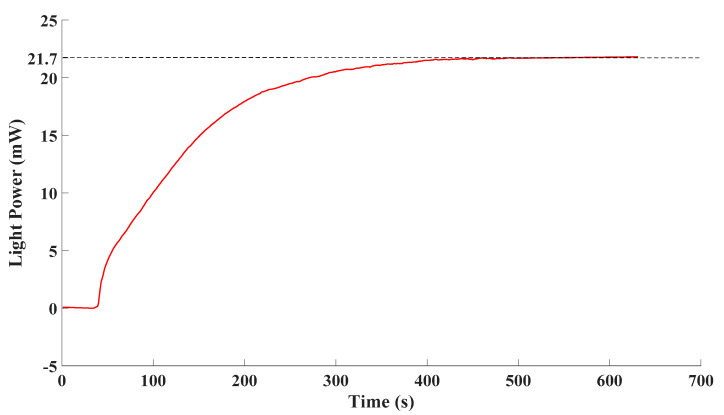
Output of the excimer lamp.

Thirdly, the drift of the proposed detection system was analyzed over time. The drift of the proposed detection system mainly came from two perspectives: the drift of the excitation and the drift of the detector. In order to evaluate their drift, the excitations of the proposed detection system, which was the excimer lamp, and the detector, which was the spectrometer, were tested respectively. Power and energy meters were employed to detect the output of the excimer lamp. The measurement results are as Figure 8.

The excimer lamp was switched on at 30 s, and after a period of ascending, the light power tended to be stable. Finally, the light power fluctuated around 21.7 mW. The standard deviation of stable data was 0.111% of the average of stable data. It can be observed that the fluctuation degree of the emission was quite small once the excimer lamp stabilized. In that case, drift of the output of excimer lamp over time could barely be observed.

On the other hand, the spectrometer was employed to measure the emission of the excimer lamp after it stabilized. Considering that every detection process often lasted less than 1 min, the emission of the stable excimer lamp for 10 successive minutes was detected. The detection results were as follows.

**Figure 9:**
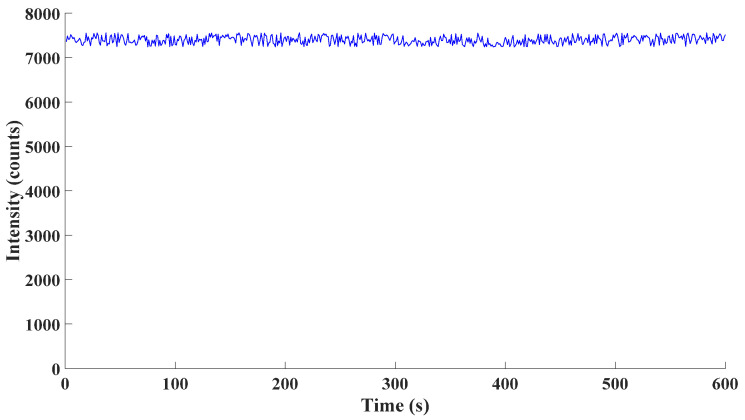
Fluctuation over time of the spectrometer.

From Figure 9, the standard deviation of experimental data was 1.22% of the average of detected data. According to experimental data, the performance of the spectrometer employed was stable over time. Hence, drift over time barely existed in the spectrometer.

In summary, the experimental results indicated that the minor fluctuation existed in the process of obtaining data. Therefore, drift over time did not emerge prominently. Such stability was beneficial for better performance of the proposed detection system.

### 4.4. Discussion and Future Works

According to above results, the feasibility of the excimer lamp in trace SO_2_ detection could be verified. On the one hand, due to the good linearity between the absorbance and the concentration of SO_2_, a gas sample with unknown concentration could be calculated via a calibrated relationship. Based on the calculation results presented in Section 4.3, it could be observed that good linearity resulted in accurate relationships and calculations. This result indicated that the proposed detection system had the capability of providing accurate detection results. On the other hand, the excimer lamp was capable in providing stable and emergent UV light as the excitation. Therefore, the emission of the excimer lamp had minor drift over time, which was beneficial to high SNR.

Based on the aforementioned experimental results, the proposed detection system possessed the DL of sub-ppm level and had fast response speed. For comparison, several common optical detection methods for trace SO_2_ are listed as follows.

From Table 4, the performance of the proposed detection system was comparable to common optical detection methods for SO_2_ such as DOAS, fluorescence, FTIR and PAS [28]. Thus, the proposed detection system has potential for practical applications.

Future studies derived from this paper may focus on two major aspects. First, due to the simple structure of proposed detection system, it was able to be highly integrated and modularized. Such a design is required when it is applied to equipment that is hard to reach, such as bushings. Second, during the operation of the microwave generator, a lot of heat was generated as well. Heat may cause the detection result to drift and the excimer lamp to quench. As a result, the heat dissipation problem is of great significance when such a detection system was implemented.

## 5. Conclusions

In this paper, a detection system for trace SO_2_ was established. Firstly, the emission characteristic of the excimer lamp was studied, and the working substance and corresponding formula of the excimer lamp were determined. Then, based on the selected excimer lamp, a UV absorption spectroscopy detection system was established for trace SO_2_. Next, the derivatives of absorbance spectra were calculated to locate the most prominent absorption peak for further linear fitting and analysis. Experimental results indicated that at the wavelength of selected absorption peak, good linearity existed between the absorbance and concentration of SO_2_. Moreover, experimental results testified the potential of a proposed detection system in trace SO_2_ quantitative measurements. Furthermore, the error budget of the proposed detection system was proposed. Based on the error budget, the detection results of the proposed detection system were quantitatively analyzed. According to the comparison the detection results and the known gas samples, the proposed detection system performed good accuracy when measuring trace SO_2_. In addition, the DL of the proposed detection system could reach a level of 10^−7^. In a nutshell, the study in this paper could verify the feasibility of the application of the excimer lamp in trace gas detection systems. The results of this paper may serve as a guideline for SF_6_-insulated equipment failure diagnosis.

## Figures and Tables

**Table 1:** Common formula and nominal wavelength of excimer lamp.

Formula	Nominal Wavelength
F-Kr	220 nm, 248 nm, 272 nm, 275 nm
Cl-Kr	200 nm, 222 nm, 240 nm, 235 nm
Br-Kr	207 nm, 222 nm, 228 nm
I-Kr	190 nm, 195 nm, 206 nm, 225 nm

**Table 2:** Accuracy of detection system.

Gas Sample	Detection Result (μL/L)	Gas Concentration (μL/L)	Relative Error (%)
1	3.9 (±23.189%)	3.3 (±2.255%)	18.1
2	6.2 (±20.239%)	6.4 (±2.255%)	3.1
3	15.5 (±7.026%)	15.8 (±2.255%)	1.9
4	84.6 (±2.023%)	83.5 (±2.255%)	1.3
5	126.3 (±1.857%)	116.9 (±2.255%)	8.0
6	139.5 (±3.393%)	135.6 (±2.255%)	2.9

**Table 3:** Systematic noise and detection limit of the detection system.

Signal of Gas Sample	Systematic Noise	Signal to Noise Ratio	Detection Limit
0.310	3.47 × 10^−3^	90.038	0.632 μL/L

**Table 4:** Comparison of the performances of common optical detection methods.

Methods	Detection Limit	Response Speed
UV-DOAS	Sub-ppm	Fast
UV fluorescence	Sub-ppm	Fast
FTIR	ppm	Medium
PAS	Sub-ppm	Fast
Proposed detection system	Sub-ppm	Fast

## Data Availability

Data sharing not applicable.
